# Extirpation of Constricted Portion of Liver

**Published:** 1888-06

**Authors:** 


### EXTIRPATION OF CONSTRICTED PORTION OF THE LIVER.

Dr. Langenbuch describes, in the *Berliner Klinische Wochenschrift*, No. 3, 1888, the case of a woman, aged 30, who had suffered for eight years from abdominal pain, most marked when she lay down. In the upper part of the abdomen a swelling could be felt. It was hardly perceptible externally, and extended from the ensiform cartilage within two and a quarter inches of the umbilical level. The swelling was smooth, tough, and elastic; its inner border was thick. Percussion impaired, the dullness being continuous with that corresponding to the area of the liver. Echinococcus or, more probably, constriction of liver, caused by clothing, was diagnosed. An exploratory incision was made, and a large mass of liver-substance, cut off by a deep constriction from the left lobe of the liver, was discovered. The mass in this case pressed the pyloric portion of the stomach, the duodenum, pancreas, aorta, with several great nerves and ganglia of the sympathetic against the vertebral column. The mass of liver was amputated, several pedicles being made out of the bands of connective tissue which still united it to the main part of the liver, and carefully ligatured. Hæmorrhage occurred on the evening of the operation; the abdominal cavity was opened up again, clots removed, and a vessel ligatured. The patient recovered for a time. Gradually ascites set in, with œdema. The abdomen was twice tapped, when the patient is said to have recovered completely.—*Exchange.*

